# Evaluation of a Decision Mining Tool for Nursing Quality Improvement by Hospital Nurses: Theory-Guided Qualitative Study

**DOI:** 10.2196/100274

**Published:** 2026-09-08

**Authors:** Matthijs Berkhout, Jobbe P L Leenen, Koen Smit, Thijs van Houwelingen

**Affiliations:** 1 Research Group Digital Ethics, University of Applied Sciences Utrecht, Heidelberglaan 15, Utrecht, Utrecht, The Netherlands, 31 621930652; 2 Faculty of Science, Open University of the Netherlands, Heerlen, Limburg, The Netherlands; 3 Research Group IT Innovations in Healthcare, Windesheim University of Applied Sciences, Zwolle, Overijssel, The Netherlands; 4 Connected Care Centre, Isala, Zwolle, Overijssel, The Netherlands; 5 Research Group Technology Use in Health and Social Care, University of Applied Sciences, Utrecht, The Netherlands

**Keywords:** decision mining, technology acceptance, quality improvement, nursing, clinical decision support, CDSS

## Abstract

**Background:**

Clinical decision support systems (CDSSs) are widely implemented in hospitals, yet their uptake in nursing practice remains inconsistent. While electronic health records continuously capture nursing decision data, these data are rarely used to support structured quality improvement. Decision mining offers a novel approach by reconstructing and visualizing decision logic from clinical decision logs to support retrospective reflection and protocol evaluation rather than real-time alerting. Before such tools can be integrated into quality improvement programs, their acceptance by clinical users must be empirically examined.

**Objective:**

This study aimed to conduct an early-stage, user-centered evaluation of a clinical decision mining tool for nursing quality improvement and to examine how nurses evaluate the factors influencing their intention to use the tool, using the unified theory of acceptance and use of technology (UTAUT) as a conceptual organizing framework.

**Methods:**

An exploratory qualitative design was used, using a theory-guided expert discussion as the primary method. A purposive sample of 5 quality improvement nurses from a large Dutch hospital participated in a 90-minute session comprising a structured prototype demonstration, a 12-item UTAUT-derived questionnaire administered as a facilitation instrument, and an expert discussion. Questionnaire scores were summarized descriptively by construct to anchor and organize the expert group discussion. Expert discussion data were analyzed using directed content analysis, with UTAUT constructs—performance expectancy, effort expectancy, attitude toward use, social influence, and behavioral intention—as sensitizing categories. Attitude toward use was included following UTAUT extensions validated in nursing informatics contexts.

**Results:**

Performance expectancy scores were consistently high, driven by perceived value for protocol evaluation and data-supported quality improvement discussions. Effort expectancy was moderate and variable, reflecting concerns about data interpretation and limited data literacy rather than interface usability. Attitude toward use showed the lowest construct-level scores and the widest variation, contingent on whether the tool was framed as learning-oriented rather than a monitoring or control mechanism. Behavioral intention was relatively high for targeted use, but participants positioned adoption in structured quality improvement meetings rather than daily in bedside practice, reflecting role differentiation rather than rejection. Social influence did not emerge as a substantive theme at this preimplementation stage.

**Conclusions:**

Nurses perceived strong value in the CDM tool’s capacity to make protocol adherence and decision variation transparent and discussable within quality improvement settings. Acceptance was conditional on a learning-oriented framing, interpretive scaffolding, and investment in data literacy. The findings suggest that governance framing and interpretive burden function as adoption determinants for retrospective decision support tools in ways not fully captured by standard UTAUT constructs. Retrospective decision mining may complement existing CDSSs not by adding alerts but by strengthening local data-driven learning cycles.

## Introduction

Clinical decision support systems (CDSSs) are increasingly used in hospitals to combine patient data with evidence-based knowledge [1,2]. Literature reviews show that CDSSs can meaningfully improve process indicators such as guideline adherence and, to a lesser extent, patient outcomes, but effects are heterogeneous and strongly context-dependent [2-5]. Despite growing interest, real-world uptake and sustained use remain problematic: many systems are underused, bypassed, or provoke workarounds rather than genuine practice change [6,7].

Although CDSS research has expanded considerably, much of the empirical evidence has focused on physician decision-making, with nursing-specific perspectives often addressed indirectly or as part of broader multidisciplinary evaluations [1,8]. Prior work has shown that the role of CDSSs in hospital nursing involves distinct tasks, workflows, and professional considerations not always captured in physician-centered evaluations [1,9]. Nurses rely on CDSSs for continuous monitoring, risk assessment, task prioritization, and care planning rather than on diagnosis alone [1]. A growing body of evidence also suggests that nurse-facing CDSSs can improve aspects of care such as glycemic control, fall prevention, and pressure injury prevention [2,8,9]. However, systematic reviews in intensive care and other high-acuity settings consistently highlight problems with usability, alert overload, and misalignment with real nursing workflows [9,10].

At the same time, hospitals have become highly data-intensive environments. Electronic health records (EHRs) and monitoring systems continuously log clinical assessments, decisions, and actions [11]. Yet, quality improvement in nursing often still relies on aggregated indicators, incident reports, and experiential judgment in team reflections rather than on systematic analysis of how decisions are made at the bedside [2,12,13]. This creates a structural paradox: digital traces of nursing decisions are routinely captured in EHRs but are rarely used to support local learning cycles or protocol evaluation.

Decision mining has been proposed to bridge this gap by using logged decision data to reconstruct, analyze, and visualize decision logic [11]. Building on design science work, Berkhout et al [11] developed and evaluated an algorithm that discovers decision structures from decision logs and visualizes them using the Decision Model and Notation (DMN) standard [14]. Rather than adding yet another real-time alerting system, the approach analyzes existing decision logs to reveal how guidelines are applied in practice, where variation occurs, and how protocols might be refined, without increasing alert burden or introducing an additional layer into daily workflows. Conceptually, such a decision mining tool could enrich quality improvement processes by making nursing decisions transparent and discussable in a format aligned with local protocols and nursing expertise.

However, existing CDSS research in nursing demonstrates that even well-designed tools can fail when they clash with nurses’ work practices, workload, or professional values [1,10,15]. A rapid review of CDSSs for hospital nurses found that benefits for decision-making, workflow efficiency, and patient outcomes are consistently moderated by alert fatigue, poor interoperability, and concerns about computational systems intruding on clinical judgment [13]. Qualitative research using the fit between individuals, tasks, and technology (FITT) framework further showed that nurses’ experiences with CDSSs are shaped by tensions between standardization and professional autonomy, between patient safety and documentation burden, and by the degree to which tools are tailored to nursing tasks rather than to medical decision-making alone [1]. These findings suggest that any novel decision-analytic tool targeting nursing quality improvement must be carefully evaluated from users’ perspectives before broader adoption.

The unified theory of acceptance and use of technology (UTAUT) provides a widely used framework for understanding such perspectives [16]. UTAUT posits that behavioral intention to use a technology is primarily driven by performance expectancy, effort expectancy, and social influence, while actual use behavior is influenced by behavioral intention and facilitating conditions. Extensions of UTAUT developed for nursing informatics contexts, such as the model by Maillet et al [17], have additionally reinstated attitude toward use as a relevant acceptance determinant, arguing that nurses’ affective and evaluative responses to clinical technologies contribute independently to adoption beyond the purely instrumental constructs of the original model. In nursing and hospital informatics, UTAUT and its extensions have been successfully applied to explain acceptance of CDSSs and related systems, with performance expectancy consistently emerging as the strongest predictor of intention to use [18].

Despite this body of work on CDSSs and decision mining, the acceptance of retrospective decision mining tools for nursing quality improvement has not yet been empirically examined. Unlike point-of-care CDSSs, retrospective decision mining tools do not intervene in real-time clinical decisions but instead generate visualizations of past decision patterns for reflection, protocol review, and improvement. They may therefore be perceived differently in terms of usefulness, required effort, perceived control, and alignment with nursing values compared to traditional alerting or recommendation systems. Given prior evidence of innovation fatigue and skepticism toward digital solutions in nursing practice, it is essential to understand how nurses evaluate such a tool before integration into quality improvement programs or departmental routines [1,11,15].

This exploratory study addresses that gap by conducting an early-stage, user-centered evaluation of a clinical decision mining (CDM) tool for nursing quality improvement from the perspective of hospital nurses. Building on the algorithmic work of Berkhout et al [11], a prototype was developed that visualizes real-world nursing decisions in DMN-based decision models and uses UTAUT and its nursing informatics extensions as the guiding framework. The study focuses on how nurses perceive the tool’s ability to support reflective practice and protocol improvement (performance expectancy), how easy the tool is to understand and work with (effort expectancy), their affective and evaluative responses to the tool (attitude toward use), and their willingness to adopt it in quality improvement contexts (behavioral intention). The following research question is addressed: “How do nurses evaluate the factors that influence their intention to use a clinical decision mining (CDM) tool for nursing quality improvement?”

## Methods

### Study Design

An exploratory qualitative design was used, using a theory-guided expert discussion as the primary method of data collection [19]. Reporting of this study followed the COREQ (Consolidated Criteria for Reporting Qualitative Research) checklist (Checklist 1) [20]. Exploratory designs are appropriate when evaluating early-stage technologies for which user experiences, contextual barriers, and implementation conditions are not yet well understood. Because decision mining represents a novel approach within nursing quality improvement, a structured prototype demonstration was included to ensure that participants could form informed judgments during the evaluation [11]. To organize and anchor the expert discussion, participants completed a brief UTAUT-based questionnaire during the discussion. The questionnaire served as a structured facilitation instrument rather than as a stand-alone measurement tool: the Likert scores generated were too limited in number to carry independent inferential weight, but they provided construct-level anchors that directed the qualitative exploration of reasoning, context, and implementation concerns. The primary analytical contribution of this study is, therefore, qualitative.

The study is explicitly formative in scope. As the prototype represents an early-stage implementation of decision mining for nursing quality improvement, the aim is to surface user acceptance signals, identify and test implementation risks, and generate early-stage design-relevant insights rather than establish definitive effectiveness or generalize findings [21].

### Prototype Description

**Figure 1.**
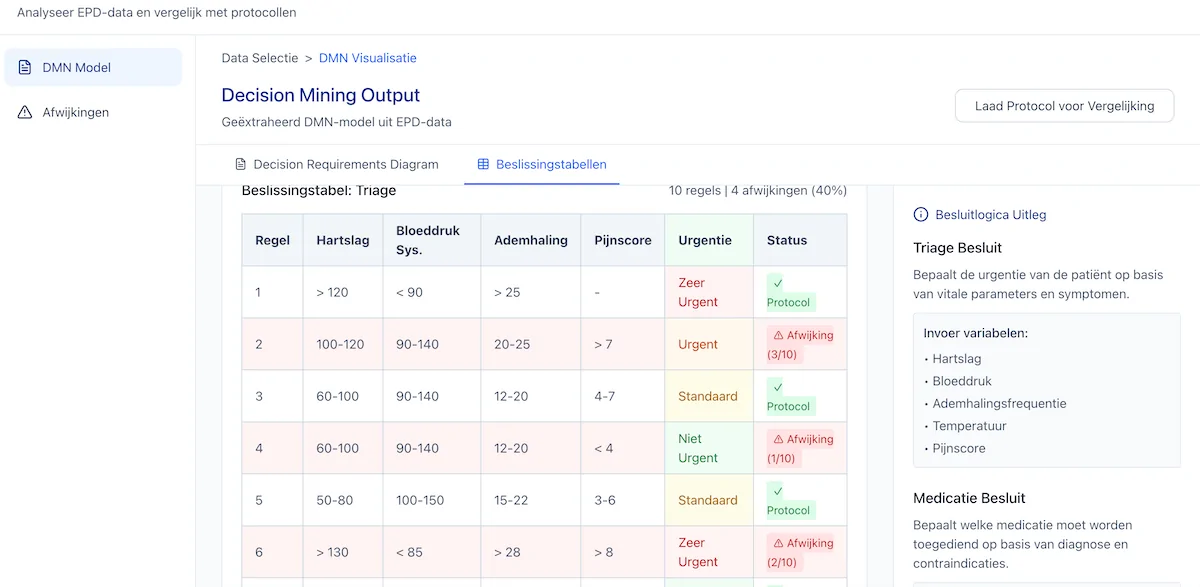
Screenshot of the clinical decision mining prototype showing a DMN decision table (in Dutch). DMN: Decision Model and Notation; EPD: *Elektronisch Patienten Dossier* (electronic health record).

The CDM prototype evaluated in this study was developed as a subsequent iteration within the same design science research program reported by Berkhout et al [11]. That prior study designed and demonstrated a fuzzy classifier algorithm capable of discovering decision structures from clinical decision logs and visualizing them using the DMN standard [14]. The algorithm operates on structured decision log data and applies fuzzy classification to identify thresholds, rules, and patterns in how decisions have been made in practice. Fuzzy classification was selected over alternative algorithms because of its interpretability and its capacity to handle continuous variables and the inherent uncertainty characteristic of clinical data; in contrast to neural network-based approaches, it produces transparent and explainable output that can be validated by subject matter experts [11]. The output consists of visualizations of clinical decision logic. For the purposes of this study, the algorithm was implemented as a prototype tool with a graphical interface to show users how to load a decision log, inspect the discovered decision structure, and navigate the resulting DMN visualization. The demonstration case used during the evaluation session was based on hospital discharge planning, a nursing-led protocol in which a structured set of clinical and logistical conditions determines whether a patient is ready for discharge. This protocol was selected in consultation with a representative of the participating hospital (JPLL) because it reflects a common decision type that is well-documented in local guidelines, making it an appropriate and recognizable use case for the evaluation. Synthetic data were used rather than real patient data to enable controlled demonstration while preserving the ecological validity of the use case. The underlying algorithmic process remained identical to the published algorithm [11]. The workflow of the prototype was presented as a working example illustrating how a decision table derived from real-world nursing discharge decisions might reveal variation in protocol adherence and serve as the basis for a quality improvement discussion (Figure 1).

Participants were informed prior to the demonstration that the data shown were synthetic and that the scenario, while realistic in structure, was constructed for evaluation purposes rather than being drawn from actual patient records at the participating hospital.

### Participants

A purposive sample of 5 quality improvement nurses (4 female, 1 male) was recruited from a large Dutch hospital. Participants were identified and approached with the assistance of a hospital representative (JPLL), who was familiar with the quality improvement teams at the participating hospital. Eligible nurses were those holding an active quality improvement role; they were invited by email or in person, and enrollment continued until 5 nurses had consented to participate. All participants were actively involved in several quality improvement initiatives, making them appropriate evaluators of a tool intended for reflective practice and protocol adherence. The sample size was justified using the information power framework proposed by Malterud et al [22], which considers sample adequacy as a function of the narrowness of the study aim, specificity of the sample, quality of dialog, and analytical strategy. In this study, the aim is narrow and construct-specific (UTAUT constructs applied to a single tool in a defined context); the sample is highly specific (nurses with active quality improvement roles, not general ward nurses); the expert discussion was structured and theoretically guided; and the analysis was deductive rather than inductive. Taken together, these conditions support the adequacy of a focused sample for the purpose of this early-stage formative evaluation, consistent with the role of pilot and feasibility studies in establishing preliminary acceptance signals before larger-scale implementation [22]. Recruitment of larger samples was not feasible due to the limited availability of nurses in these specialized quality improvement roles, a challenge frequently noted in studies involving quality-focused clinical personnel [23].

### Procedure

Data collection comprised 2 sequential phases within a 90-minute session conducted at the participating hospital, in a meeting room on-site, during or directly after the participants’ working hours. The session was facilitated by a second researcher, KS, a coauthor without direct involvement in the prototype development, to reduce the reflexive risk associated with the first author’s dual role as both developer and lead researcher. The first author, MB, was present to deliver the prototype demonstration, respond to technical questions, and lead the structured expert discussion. In the first phase (approximately 15 minutes), participants attended a structured demonstration of the CDM prototype, which illustrated how EHR decision log data can be transformed into decision models and DMN visualizations. Because decision mining is unfamiliar to most nurses, a structured prototype demonstration was included so that participants could form informed judgments during the evaluation. To ensure ecological validity, the demonstration case embedded in the prototype was reviewed in advance with a representative of the participating hospital (JPLL). Although synthetic data were used, the scenario and decision logic were aligned with local workflows and nursing practices, enabling participants to evaluate the tool based on a realistic and recognizable use case.

In the second phase (approximately 75 minutes), participants completed a 12-item questionnaire drawing on specific UTAUT-based instrumentation [16,17]. The instrument measured performance expectancy, effort expectancy, social influence, and behavioral intention, consistent with core UTAUT-derived constructs, as well as attitude toward use, which was included following UTAUT extensions validated in nursing informatics contexts that reinstate attitude as a relevant determinant of acceptance [17]. As operationalized in this study, performance expectancy refers to the degree to which a nurse believes that using the CDM tool would improve their quality improvement and protocol-evaluation work. Effort expectancy refers to the degree of ease associated with learning and using the tool. Attitude toward use refers to a nurse’s overall affective and evaluative response to using the tool, and behavioral intention refers to the strength of a nurse’s intention to use the tool within quality improvement activities [16,17]. Social influence is defined as the degree to which a nurse perceives that important others believe they should use the tool. Items used 7-point Likert scales (1=“fully disagree” and 7=“fully agree”). The questionnaire is available in Multimedia Appendix 1, and the full session protocol is available in Multimedia Appendix 2. The items were administered one at a time: after each item, participants briefly recorded their individual score, and the facilitator then opened the floor for discussion of that item before moving on to the next. In this way, the questionnaire functioned as a structured facilitation instrument that anchored an item-by-item expert discussion rather than as a stand-alone survey, a format well-suited for eliciting collective reflections, shared reasoning, and emerging concerns about new technologies in health care [24,25]. The open questions during the expert discussion explored the reasoning behind questionnaire scores, enabling participants to articulate the conditions, concerns, and contextual factors underlying their responses. The discussion was audio-recorded and transcribed verbatim for analysis.

### Data Analysis

The questionnaire data were summarized descriptively. For each item, the median and range were calculated to characterize the spread of responses across participants, and construct-level summaries were derived by aggregating item scores within each UTAUT dimension. They are reported here for contextual reference rather than as inferential findings.

Expert discussion data were analyzed using directed content analysis [26], a theory-informed approach in which predefined categories derived from an existing framework guide the coding and interpretation of textual data. The UTAUT-derived constructs—performance expectancy, effort expectancy, and behavioral intention—served as primary sensitizing categories, supplemented by attitude toward use, which was included as an additional category following UTAUT extensions that reinstate attitude as a relevant category in nursing informatics acceptance research [17]. Although social influence was included in the questionnaire, it generated minimal discussion in the expert discussion and is therefore not reported as a substantive finding; this absence is noted as a limitation. Coding was performed using Microsoft Excel. The verbatim transcript was first read in full to gain familiarity, after which segments were coded and assigned to constructs by MB, with findings reviewed and discussed by the broader author team. The expert discussion transcript was examined construct by construct to identify explanatory patterns that clarified how participants understood and justified their questionnaire responses. These patterns included, for example, conditional perceptions of usefulness, distinctions between individual and team-level adoption, and contextual factors shaping behavioral intentions. This analytic strategy aligns with qualitative approaches that focus on explaining variation in theoretically defined categories rather than generating new theory [27]. Illustrative quotations were selected to demonstrate how qualitative insights contextualized the score patterns. To strengthen trustworthiness, several procedures were applied: the structured, theory-guided interview guide supported dependability and independent probing by the co-facilitator (KS) supported credibility and confirmability. Verbatim quotations from multiple participants are presented in the “Results” section to allow readers to assess the grounding of the findings.

### Reflexivity Statement

The first author is also the principal developer of the CDM prototype evaluated in this study, as part of a broader design science research program. This dual role creates a potential for positive framing or selective emphasis in both the demonstration and the interpretation of findings. To mitigate this, several procedural steps were taken. The demonstration script was prepared in advance and reviewed by a hospital representative, JPLL, and the coauthors KS and TVH prior to the session. The expert discussion was co-facilitated by a second researcher KS, a coauthor not directly involved in prototype development, who participated in the discussion and probed for concerns and critical responses independently of the first author. Participants were explicitly informed that the prototype is an early-stage research artifact and were encouraged to express reservations and critique alongside positive evaluations. The analytic process was reviewed and, where needed, discussed by the full author team before conclusions were finalized. These procedures do not eliminate reflexive risk but make the conditions of the study transparent.

### Ethical Considerations

Ethical approval was obtained from the Ethics Review Board of HU University of Applied Sciences Utrecht (ref: 357-001-2025). Participation was voluntary, and written informed consent was obtained from all participants prior to data collection. To protect privacy and confidentiality, all data were deidentified, with participants referred to only by codes (P1-P5), and the expert discussion transcript was stored securely on institutional servers with access restricted to the research team. The study used synthetic demonstration data, so no real patient data were processed at any stage. Participants received no compensation for their involvement.

## Results

This section presents the descriptive questionnaire scores for each UTAUT construct, followed by directed content analysis findings that explain and contextualize the scoring patterns. The Likert scale summaries are included for transparency and as a contextual reference for the qualitative findings, not as stand-alone quantitative results. Participant quotations are identified by participant codes (P1-P5).

### Performance Expectancy

Performance expectancy concerns whether nurses expect the tool to improve their quality improvement work. Responses were consistently high across items, indicating a strong shared expectation that the CDM tool would add value to clinical decision-making and quality improvement for nurses (Table 1). These high scores are reflected in respondents’ emphasis on moving from experiential reasoning to data-supported action. Decisions about protocols were described as being currently based on repeated experience: “You discuss it and think, well, these points should be adjusted in the protocol, because you have encountered it a few times” (P2). The availability of data was perceived as enabling more explicit and actionable discussions: “If you are going to discuss certain issues, for example addressing a protocol, you should also make sure that it is clearly visible in your data, so that you can actually act upon it” (P2).

**Table 1.** Performance expectancy questionnaire scores by participants.

Item	Question	P1	P2	P3	P4	P5
Q1	Using the decision mining tool would increase my effectiveness in clinical decision-making.	7	5	6	6	5
Q2	Using the tool would improve the quality of patient care.	5	6	7	7	6
Q3	The decision mining tool would be useful in my daily work.	5	6	7	6	6

Respondents linked effectiveness to increased precision in clinical thresholds, which was seen as leading to concrete improvements such as fewer alerts: “But if you have data, you can be very precise, it does not have to be 140 over 80; it could perhaps be 138 over, just as an example, you can adjust it very accurately. As a result, you have fewer alerts” (P2). High scores were further explained by the perceived ability to justify decisions to others: “And thus to be able to substantiate [the decisions] as well” (P1). Perceived usefulness was also grounded in concrete quality-of-care examples. Respondents described how data were used to support changes in pain assessment practices without compromising patient outcomes: “At a certain point, we demonstrated here that people no longer experience unacceptable pain if you only assess pain scores when there is an indication” [...] “That was supported by data” (P1).

In addition, respondents associated usefulness with workload reduction: “Yes, and that is also very practical in terms of reducing workload” (P2) and “That is also very practical in daily practice, in terms of workload reduction. That’s when it becomes interesting, like, something has actually changed if you look at my work process. And when data supports that it isn’t necessary, that means we’ve been doing unnecessary work” (P3). Despite these (high) scores, respondents indicated that the perceived value mainly applied to evaluation and improvement rather than day-to-day clinical steering: “It [the prototype] remains somewhat limited to looking back, rather than guiding day-to-day practice” (P2). This statement also explains why performance expectancy scores were high but not uniformly at the highest score.

### Effort Expectancy

Effort expectancy concerns the perceived ease of learning and using the tool. Responses were moderate and varied more widely across participants than those for performance expectancy, indicating mixed expectations regarding the ease of learning and using the CDM tool (Table 2). Respondents indicated that they would hesitate to use the tool without first understanding what the data represent, which helps explain why ease-of-use ratings did not reach the high levels observed for perceived usefulness: “You wonder what you are actually looking at, and then I won’t immediately use it during a quality improvement review, because first I want to understand myself what I am looking at” (P3).

**Table 2.** Effort expectancy questionnaire scores by participants.

Item	Question	P1	P2	P3	P4	P5
Q4	Learning to work with the decision mining tool would be easy.	3	5	6	N/A^a^	6
Q5	The tool feels user-friendly.	5	6	5	6	4
Q6	I could quickly become skilled at using the tool.	3	6	6	4	5

a N/A: not applicable.

Effort expectancy was further tempered by expectations of interaction complexity and cognitive load: “I think it would cause total panic for everyone, including myself. Because it still involves quite a bit of clicking” and “You really have to interpret it” (P5). These expectations were not viewed as specific to the CDM tool but as consistent with prior experiences with existing dashboards: “And that is also how the dashboards we currently have are used” (P5). Finally, respondents linked their anticipated difficulty in becoming proficient to broader limitations in data literacy within the nursing workforce, which helped explain both the moderate median score and the relatively large spread in responses: “That data-driven way of working really isn’t there. It’s only a very small group” (P1) and “They can already panic over Excel” (P5). Taken together, these findings indicate that effort expectancy is constrained less by the perceived design of the tool itself and more by uncertainty about data interpretation skills and prior experiences with complex data environments.

### Attitude Toward Use

Attitude toward use concerns nurses’ overall affective and evaluative response to the tool. Responses to the attitude items were generally moderate and revealed a conditional acceptance of the tool. Participants did not reject the concept itself; rather, their attitudes depended strongly on how the tool would be framed and implemented in practice. A recurring theme was the distinction between learning-oriented and control-oriented use (Table 3). Acceptance decreased when the tool was perceived as a monitoring mechanism: “If we start monitoring with it as well, it becomes less effective*”* (P5). This suggests that positive attitudes are contingent upon positioning the tool as supportive and reflective rather than evaluative or punitive. Participants also emphasized the importance of clarity of purpose. Without a clearly defined objective, enthusiasm decreased: “The purpose must be clear: why are you using that tool?” (P3). Importantly, resistance was not directed toward technology itself but toward the complexity of the underlying data: “It is not the tool itself; it is the complexity of the data” (P5).

**Table 3.** Attitude toward use questionnaire scores by participants.

Item	Question	P1	P2	P3	P4	P5
Q7	I have a positive attitude toward using the decision mining tool in my work.	4	4	6	6	5
Q8	Using the tool seems like a good idea to me.	1	3	3	2	2
Q9	I enjoy working with new technologies such as this tool.	1	3	5	4	3

### Behavioral Intention

Behavioral intention concerns the strength of nurses’ intention to use the tool in quality improvement activities. Responses reflected selective rather than universal willingness to use the tool (Table 4). Participants did not envision broad, routine adoption by all nurses, but rather targeted use by those actively involved in quality improvement activities: “I don’t think everyone should have to work with it, but the people who are involved in quality improvement could definitely benefit a lot from it” (P5). Intended use was primarily situated within structured quality meetings rather than daily bedside practice: “I can definitely see this being used in quality improvement meetings, but it’s not something you would use to steer day-to-day practice” (P2). These statements indicate that participants perceive the tool as appropriate for reflective, periodic analysis rather than continuous clinical decision support. The moderate intention scores, therefore, appear to reflect role differentiation and contextual use, rather than rejection of the tool itself.

**Table 4.** Behavioral intention questionnaire scores by participants.

Item	Question	P1	P2	P3	P4	P5
Q10	If this tool becomes available, I intend to use it in my work.	5	3	5	6	5
Q11	I expect to use the decision mining tool regularly in the future.	7	5	6	7	5
Q12	I would recommend this tool to colleagues.	7	6	6	7	5

## Discussion

### Principal Findings

This exploratory qualitative study examined how quality improvement nurses evaluate a CDM tool intended to support nursing quality improvement, using UTAUT as a conceptual organizing framework to structure both data collection and analysis. Across UTAUT constructs, a consistent and interpretable pattern emerged. Performance expectancy was high, driven by perceived value for data-supported protocol evaluation rather than real-time clinical steering. Effort expectancy was moderate and variable, reflecting concerns about data literacy and interpretive burden in the nursing workforce rather than interface usability. Attitude toward use was the lowest and most variable construct, contingent on whether the tool was framed as learning-oriented rather than controlling. Behavioral intention was relatively high but selectively positioned within quality improvement meetings and quality-focused nursing roles rather than across all nursing functions. Together, these findings characterize the CDM tool as a contextually promising but conditionally accepted innovation whose uptake depends primarily on organizational framing, data literacy infrastructure, and clarity of purpose.

### Performance Expectancy: Retrospective Value in a Prospective Field

The high performance expectancy scores align with the CDSS literature, which demonstrates that perceived usefulness is the dominant predictor of technology acceptance, particularly when tools provide actionable, workflow-relevant benefits [16,28]. Importantly, the CDM tool was evaluated as a retrospective analysis instrument rather than a real-time decision support system, and participants framed its value primarily in terms of improving the quality and precision of protocol revision discussions. This learning-oriented positioning may address known barriers to real-time CDSSs, particularly alert burden and workflow disruption, which are repeatedly highlighted in nursing-focused systematic reviews [9,10]. It also aligns with the broader CDSS evidence base, which shows that system success depends strongly on how tools are embedded in local work practices and improvement routines rather than on functionality alone [3-5]. For clinical practice and implementation, this positioning matters: a retrospective tool that enriches periodic protocol review meetings occupies a different and potentially less contested space in nursing workflows than a system that intervenes in real-time decisions.

### Effort Expectancy: Interpretive Burden as the Core Barrier

Effort expectancy was moderate and variable. Qualitative explanations consistently pointed to data interpretation rather than interface complexity as the primary source of anticipated effort. This finding is consistent with implementation research showing that adoption challenges frequently arise from sociotechnical factors such as skill gaps, support structures, and fit with existing routines rather than from technology characteristics alone [15,29]. The participants’ repeated emphasis on needing first to understand what they are looking at before using the tool in a professional context suggests that CDM requires a data literacy threshold that many nurses in the current workforce have not yet met. This insight connects to a broader challenge in health informatics: the gap between data availability and the capability to interpret and act on clinical data in practice [17]. In UTAUT terms, a high performance expectancy combined with moderate effort expectancy creates a specific implementation tension: the tool is perceived as valuable, but readiness to engage is constrained by limited confidence in data handling [16,18]. For CDM, this implies that data literacy and interpretive scaffolding are not supplementary concerns but rather core enabling conditions for adoption.

### Attitude Toward Use: Framing as an Adoption Determinant

Attitude toward use showed the lowest construct median and the most pronounced internal variation, particularly in the very low scores on Q8. The qualitative data reveal that this is not a reflection of technological skepticism but of sensitivity to governance framing. Acceptance was contingent on whether the tool was positioned as a learning and protocol-refinement instrument rather than as a performance-monitoring or control mechanism. This resonates with qualitative evidence that CDSS uptake is consistently undermined when technologies are perceived as threatening professional judgment or increasing administrative surveillance [13,15]. For CDM specifically, the implication is that organizational framing is not cosmetic but constitutive of adoption. The same tool, presented with different institutional narratives, is likely to generate substantially different acceptance profiles. Positioning CDM explicitly as a shared learning instrument for quality teams, rather than as a management or compliance tool, may therefore be a necessary condition for avoiding the defensive responses and workarounds documented elsewhere for poorly framed CDSSs [6,7].

### Behavioral Intention: Role Differentiation Rather Than Rejection

Behavioral intention was relatively high, but participants consistently positioned adoption as role-specific and meeting-centered rather than universal and routine. This pattern should not be misread as a partial rejection of the CDM tool. Prior CDSS research shows that sustained uptake depends on credible task-technology fit and that systems are frequently underused when deployed without a clearly defined and contextually appropriate use case [6,7]. In this study, participants articulated a clear and coherent use case for the CDM tool within structured quality improvement meetings conducted by quality-focused nurses. This alignment between intended tool purpose and perceived appropriate use context is, arguably, a more favorable adoption signal than uniformly high intention scores that prove contextually unsustainable in practice.

### Implications for Design and Implementation

The findings have 4 interconnected implications for CDM tool development and deployment. First, embedding CDM within recurring quality improvement routines, such as protocol review meetings, aligns with how participants naturally framed the tool’s value and is likely to support rather than disrupt nursing workflows. Second, providing interpretive scaffolding through worked examples, narrative explanations, and guided walkthroughs will be essential to reduce the cognitive load associated with data interpretation, which participants identified as the primary effort barrier. Third, investing in data literacy at the organizational level is a prerequisite for effective CDM adoption, as effort expectancy barriers were tied to broader workforce capabilities rather than to tool design alone, a finding consistent with prior nursing CDSS implementation literature [1,17]. Fourth, and perhaps most critically, organizations must frame CDM use explicitly as a learning and improvement instrument rather than as a monitoring or accountability mechanism, given that this framing distinction was the primary driver of conditional acceptance and the likely trigger for professional resistance if mishandled.

### Limitations

This study has several limitations that should be acknowledged transparently. The sample was small (N=5) and purposively selected from nurses with active quality improvement roles in a single Dutch hospital, which supports domain relevance but substantially limits generalizability to other nursing populations, hospital contexts, and health systems. The evaluation used a prototype demonstrated with synthetic rather than real local data. This is particularly relevant to the interpretation of performance expectancy scores: participants evaluated the tool’s potential based on a constructed scenario aligned with their practice, but they never encountered the interpretive challenges that would arise from actual, messy, locally familiar clinical decision data. Performance expectancy scores may therefore be optimistically inflated relative to what would emerge under real deployment conditions, and the effort expectancy concerns about data interpretation, which participants themselves raised, may be even more prominent in practice than the current findings suggest. A related constraint is that the demonstration, questionnaire completion, and expert discussion were compressed into a single 90-minute session. In addition, social influence was assessed with a single item (Q12) connected to behavioral intention in contrast to the multi-item coverage of the other constructs, and it generated minimal discussion during the expert session. Therefore, it could not be reported as a substantive finding, and its role in this context remains unexamined. These limitations are consistent with the role of early-stage formative evaluation studies, which aim to surface acceptance signals and implementation risks rather than to establish definitive or generalizable effects.

### Future Research

Future research should investigate how the CDM tool functions over time in real-world quality improvement processes across diverse hospital contexts and nursing populations. Longitudinal studies are needed to examine whether repeated use in structured reflection sessions reduces interpretive barriers and builds the data literacy required for effective adoption. Participants highlighted concerns about data quality in EHRs; future work should therefore examine how decision mining can be leveraged to identify documentation inconsistencies and to stimulate improvements in organizational data governance practices. Further design iterations should prioritize interpretability and accessibility for nurses across varying levels of digital literacy, including the evaluation of different visualization approaches and scaffolding strategies. Finally, studies comparing acceptance profiles across different nursing specialties and health care systems will be necessary to understand the boundary conditions of the findings reported here.

### Conclusions

This exploratory study examined how nurses engaged in quality improvement evaluate a CDM tool using the UTAUT as the guiding framework. Nurses perceived strong value in the tool’s capacity to make protocol adherence and decision variation transparent and discussable within structured quality improvement settings. However, acceptance was conditional across multiple dimensions: effort expectancy was shaped primarily by concerns about data interpretation and workforce data literacy rather than interface complexity; attitude toward use depended strongly on whether the tool was positioned as a learning instrument rather than a monitoring mechanism; and behavioral intention was selectively anchored to quality improvement roles and meeting contexts rather than to routine bedside practice.

These findings have theoretical and practical implications. Theoretically, they suggest that UTAUT constructs manifest differently in retrospective decision support tools than in point-of-care CDSSs, with framing and governance emerging as particularly prominent determinants of attitude and intention. Practically, successful implementation of CDM tools requires integration into existing quality improvement routines, investment in data literacy and interpretive support, and deliberate and transparent learning-oriented framing at the organizational level. Retrospective decision mining may complement existing CDSSs not through additional real-time alerts but by strengthening the local data-driven learning cycles that are necessary for sustainable protocol improvement in nursing practice.

## Supplementary material

10.2196/100274 Multimedia Appendix 1 Questionnaire for focus group.

10.2196/100274 Multimedia Appendix 2 Session protocol translated in English.

10.2196/100274 Checklist 1 COREQ checklist.

## Abbreviations

CDM: clinical decision mining
CDSS: clinical decision support system
COREQ: Consolidated Criteria for Reporting Qualitative Research
DMN: Decision Model and Notation
EHR: electronic health record
FITT: fit between individuals, tasks, and technology
UTAUT: Unified Theory of Acceptance and Use of Technology
