# Supplementary material for: Evaluation of a Decision Mining Tool for Nursing Quality Improvement by Hospital Nurses: Theory-Guided Qualitative Study
Source: JMIR Form Res. 2026 Sep 8;10:e100274. doi: 10.2196/100274 (PMC13552989; doi:10.2196/100274)
Supplement: Supplementary file 1 [file formative-v10-e100274-s001.pdf]

UTAUT based Questionaire:

Vraag	Construct	Toelichting & nut	Question (English)
**1. Het gebruik van de decision-mining-tool zou mijn effectiviteit bij klinische beslissingen vergroten.**	Performance expectancy	Evalueert of verpleegkundigen verwachten dat de tool hen helpt betere beslissingen te nemen; dit is één van de sterkste voorspellers van gebruiksintentie .	1. Using the decision mining tool would increase my effectiveness in clinical decision-making.
**2. Het gebruik van de tool zou de kwaliteit van de patiëntenzorg verbeteren.**	Performance expectancy	Meet de verwachte impact op zorguitkomsten; nuttig om te zien of verpleegkundigen de toegevoegde waarde van de tool voor de patiënt herkennen.	2. Using the tool would improve the quality of patient care.
**3. De decision-mining-tool zou nuttig zijn in mijn dagelijkse werk.**	Performance expectancy	Algemeen nut voor het takenpakket; helpt om te bepalen of de tool relevant wordt geacht voor routine-taken.	3. The decision mining tool would be useful in my daily work.
**4. Het leren werken met de decision-mining-tool zou gemakkelijk zijn.**	Effort expectancy	Meet de verwachte leercurve; volgens UTAUT beïnvloedt gebruiksgemak direct de intentie om een systeem te gebruiken .	4. Learning to work with the decision mining tool would be easy.
**5. De tool voelt gebruiksvriendelijk aan.**	Effort expectancy	Vraagt naar de algemene ervaren gebruiksgemak na de demo; relevant om praktische obstakels te identificeren.	5. The tool feels user-friendly.

**6. Ik zou snel vaardig kunnen worden in het gebruik van de tool.**	Effort expectancy	Toetst of verpleegkundigen vertrouwen hebben dat ze de tool zonder veel moeite kunnen toepassen.	6. I could quickly become skilled at using the tool.
**7. Ik sta positief tegenover het gebruik van de decision-mining-tool in mijn werk.**	Attitude towards use	Meet de algemene houding; attitude is een sleutelconstruct in TAM en beïnvloedt de intentie om te gebruiken.	7. I have a positive attitude toward using the decision mining tool in my work.
**8. Het gebruiken van de tool lijkt mij een goed idee.**	Attitude towards use	Evalueert of de technologie als waardevol gezien wordt; sluit aan bij de focusgroepdiscussie over perceptie.	8. Using the tool seems like a good idea to me.
**9. Ik vind het prettig om met nieuwe technologieën te werken zoals deze tool.**	Attitude towards use	Toetst technologische openheid; kan verschillen verklaren tussen ervaren en minder ervaren verpleegkundigen.	9. I enjoy working with new technologies such as this tool.
**10. Als deze tool beschikbaar is, ben ik van plan om deze in mijn werk te gebruiken.**	Behavioral intention	Vraagt direct naar de toekomstige gebruiksintentie; sterk gerelateerd aan daadwerkelijk gebruik.	10. If this tool becomes available, I intend to use it in my work.
**11. Ik verwacht de decision-mining-tool regelmatig te gebruiken in de toekomst.**	Behavioral intention	Kwantificeert hoe frequent men de tool wil inzetten; nuttig voor inschatting van adoptie.	11. I expect to use the decision mining tool regularly in the future.
**12. Ik zou deze tool aanbevelen aan collega's.**	Behavioral intention	Meet bereidheid tot aanbeveling, wat een indicatie is van tevredenheid en	12. I would recommend this tool to colleagues.

		verwachte sociale invloed binnen het team.	
