# Supplementary material for: Evaluation of a Decision Mining Tool for Nursing Quality Improvement by Hospital Nurses: Theory-Guided Qualitative Study
Source: JMIR Form Res. 2026 Sep 8;10:e100274. doi: 10.2196/100274 (PMC13552989; doi:10.2196/100274)
Supplement: Supplementary file 2 [file formative-v10-e100274-s003.pdf]

COREQ (COnsolidated criteria for REporting Qualitative research) Checklist

Manuscript: How Nurses Evaluate a Decision Mining Tool for Quality Improvement: A Theory-Guided Qualitative Study

Topic	Item No.	Guide Questions / Description	Reported on Page No.
**Domain 1: Research team and reflexivity**			
**Personal characteristics**			
Interviewer / facilitator	1	Which author/s conducted the interview or focus group?	p. 7
Credentials	2	What were the researcher's credentials? E.g. PhD, MD	p. 1 (affiliations)
Occupation	3	What was their occupation at the time of the study?	p. 1 (affiliations); p. 8
Gender	4	Was the researcher male or female?	N/A
Experience and training	5	What experience or training did the researcher have?	p. 8
**Relationship with participants**			
Relationship established	6	Was a relationship established prior to study commencement?	p. 7; p. 8
Participant knowledge of the interviewer	7	What did the participants know about the researcher? e.g. personal goals, reasons for doing the research	p. 8
Interviewer characteristics	8	What characteristics were reported about the interviewer/facilitator? e.g. bias, assumptions, reasons and interests in the research topic	p. 8
**Domain 2: Study design**			
**Theoretical framework**			
Methodological orientation and theory	9	What methodological orientation was stated to underpin the study? e.g. grounded theory, discourse analysis, ethnography, phenomenology, content analysis	p. 5; p. 8
**Participant selection**			
Sampling	10	How were participants selected? e.g. purposive, convenience, consecutive, snowball	p. 6
Method of approach	11	How were participants approached? e.g. face-to-face, telephone, mail, email	p. 6
Sample size	12	How many participants were in the study?	p. 2 (abstract); p. 6
Non-participation	13	How many people refused to participate or dropped out? Reasons?	p. 6
**Setting**			
Setting of data collection	14	Where was the data collected? e.g. home, clinic, workplace	p. 7
Presence of non-participants	15	Was anyone else present besides the participants and researchers?	p. 7; p. 8

Topic	Item No.	Guide Questions / Description	Reported on Page No.
Description of sample	16	What are the important characteristics of the sample? e.g. demographic data, date	p. 6
**Data collection**			
Interview guide	17	Were questions, prompts, guides provided by the authors? Was it pilot tested?	p. 7; p. 16 (data availability)
Repeat interviews	18	Were repeat interviews carried out? If yes, how many?	N/A — single session
Audio/visual recording	19	Did the research use audio or visual recording to collect the data?	p. 7
Field notes	20	Were field notes made during and/or after the interview or focus group?	p. 7
Duration	21	What was the duration of the interviews or focus group?	p. 2 (abstract); p. 7
Data saturation	22	Was data saturation discussed?	p. 15
Transcripts returned	23	Were transcripts returned to participants for comment and/or correction?	p. 8
**Domain 3: Analysis and findings**			
**Data analysis**			
Number of data coders	24	How many data coders coded the data?	p. 8; p. 15
Description of the coding tree	25	Did authors provide a description of the coding tree?	p. 8
Derivation of themes	26	Were themes identified in advance or derived from the data?	p. 8
Software	27	What software, if applicable, was used to manage the data?	p. 8
Participant checking	28	Did participants provide feedback on the findings?	p. 8
**Reporting**			
Quotations presented	29	Were participant quotations presented to illustrate the themes/findings? Was each quotation identified? e.g. participant number	pp. 9-12
Data and findings consistent	30	Was there consistency between the data presented and the findings?	pp. 9-15
Clarity of major themes	31	Were major themes clearly presented in the findings?	pp. 9-12; p. 12
Clarity of minor themes	32	Is there a description of diverse cases or discussion of minor themes?	pp. 9-12; p. 15

Developed from: Tong A, Sainsbury P, Craig J. Consolidated criteria for reporting qualitative research (COREQ): a 32-item checklist for interviews and focus groups. International Journal for Quality in Health Care. 2007. Volume 19, Number 6: pp. 349-357
