# Supplementary material for: Evaluation of a Decision Mining Tool for Nursing Quality Improvement by Hospital Nurses: Theory-Guided Qualitative Study
Source: JMIR Form Res. 2026 Sep 8;10:e100274. doi: 10.2196/100274 (PMC13552989; doi:10.2196/100274)
Supplement: Supplementary file 3 [file formative-v10-e100274-s002.docx]

# Session protocol

# Procedure:

***0:00–0:10 Opening, introduction & context***

“Thank you all for being here. Today we are testing a digital tool that can automatically visualize nursing decisions from the EHR data. The aim is to investigate whether this can help with quality improvement and team reflection. We are not concerned with individual performance, but with patterns that we can see as a team.

***0:10–0:15 Brief explanation of the tool and DMN***

The digital tool uses a standardized way of representing decisions, called DMN (Decision Model and Notation). You can think of DMN as:

- A visual representation of how a decision is normally made,
- A diagram showing which information you need (e.g. blood pressure, pain score, medication),
- And a table showing which outcome corresponds to which combination of data.

It resembles a step-by-step plan or flowchart, but much clearer and always following the same structure. This allows you to:

- Easily see whether practice matches the protocol,
- Explain why certain decisions differ,
- And discuss together, in a focused way, where quality improvement is possible.

***0:15–0:20 Scenario 1: Missed sub-decision in the discharge decision***

*Time for clarifying questions about the case.*

***0:20–0:35 Scenario 2: Deviations at the decision-rule level***

**Scenario 2.1 Deviation in a simple decision rule**

**Scenario 2.2 Larger scale: 25% deviations across 2,500 patients**

*Time for clarifying questions about the case.*

***0:35–1:15 Discussion of UTAUT questions***

- Have participants fill in the questions live.
- Show the charts directly on screen.
- Address the extremes briefly for each question:
  - “I see that some people strongly agree/disagree. Who would like to explain what lies behind that?”
  - “Can you explain your choice?”

***General questions to ask:***

***1:15–1:25 General discussion and closing questions***

- Is there anything we have not discussed today that you consider important for the use or development of this digital tool?
- What would be the most important thing for us to take into account if we want to further improve or implement this digital tool?
- Are there conditions or peripheral matters that, in your view, need to be right before such a digital tool is successful in your daily work?
- How does this tool fit the way you like to work?
- How do you see this tool potentially becoming part of quality discussions or team reflections in the future?
- Are there situations in your work where this tool would specifically not be appropriate?

**1:25–1:30 Closing**
